# Halloysite- and Montmorillonite-Loaded Scaffolds as Enhancers of Chronic Wound Healing

**DOI:** 10.3390/pharmaceutics12020179

**Published:** 2020-02-20

**Authors:** Giuseppina Sandri, Angela Faccendini, Marysol Longo, Marco Ruggeri, Silvia Rossi, Maria Cristina Bonferoni, Dalila Miele, Adriele Prina-Mello, Carola Aguzzi, Cesar Viseras, Franca Ferrari

**Affiliations:** 1 Department of Drug Sciences, University of Pavia, Viale Taramelli 12, 27100 Pavia, Italy; angela.faccendini@gmail.com (A.F.); marysol.longo01@universitadipavia.it (M.L.); marco.ruggeri02@universitadipavia.it (M.R.); silvia.rossi@unipv.it (S.R.); cbonferoni@unipv.it (M.C.B.); dalila.miele@gmail.com (D.M.); franca.ferrari@unipv.it (F.F.); 2 Trinity Translational Medicine Institute, Trinity College Dublin, Dublin 8, Dublin, Ireland; prinamea@tcd.ie; 3 Department of Pharmacy and Pharmaceutical Technology, Faculty of Pharmacy, University of Granada, Campus of Cartuja s/n, 18071 Granada, Spain; carola@ugr.es (C.A.); cviseras@ugr.es (C.V.)

**Keywords:** electrospinning, chitosan, chondroitin sulfate, scaffolds, montmorillonite, halloysite, fibroblasts proliferation, immune response

## Abstract

The increase in life expectancy and the increasing prevalence of diabetic disease and venous insufficiency lead to the increase of chronic wounds. The prevalence of ulcers ranges from 1% in the adult population to 3–5% in the over 65 years population, with 3–5.5% of the total healthcare expenditure, as recently estimated. The aim of this work was the design and the development of electrospun scaffolds, entirely based on biopolymers, loaded with montmorillonite (MMT) or halloysite (HNT) and intended for skin reparation and regeneration, as a 3D substrate mimicking the dermal ECM. The scaffolds were manufactured by means of electrospinning and were characterized for their chemico-physical and preclinical properties. The scaffolds proved to possess the capability to enhance fibroblast cells attachment and proliferation with negligible proinflammatory activity. The capability to facilitate the cell adhesion is probably due to their unique 3D structure which are assisting cell homing and would facilitate wound healing in vivo.

## 1. Introduction

The skin is the largest organ of the body and plays a pivotal role in maintaining physiological homeostasis against fluid imbalance, thermal dysregulation, and infections. It is formed by the epidermis, consisting of keratinocytes, and by the dermis, mainly based on the extracellular matrix (ECM) (collagen, elastin, glycosaminoglycans) and sparse fibroblasts. Damage or loss of integrity of the skin caused by a wound, may impair skin functions, exposing the body to potentially challenging situations. Wound healing is a complex event based on overlapping but well-orchestrated cellular and molecular processes to repair damaged tissue and restore skin function. Healing is divided in different phases (hemostasis, inflammatory, proliferative, and remodeling) and is accomplished by ECM molecules, soluble mediators, as cytokines and growth factors, various resident cells, and infiltrating leucocytes [1].

Acute wounds are mainly traumatic or surgical and generally heal within few weeks without any significant interventions, whereas in chronic wounds, the healing process remains frozen in the inflammatory state. These, commonly defined as wounds that fail to proceed through an orderly and timely process to restore skin anatomical and functional integrity. These include venous leg ulcers, arterial ulcers, diabetic ulcers, and pressure ulcers, such as bed sores. The increase in life expectancy and the increasing prevalence of diabetic disease and venous insufficiency lead to an increase in chronic wounds. Although assessing the prevalence of chronic wounds is problematic because of the disparities in study design and their evaluation, they have become a major challenge to healthcare systems worldwide. It is estimated that the prevalence of ulcers ranges from 1% in the adult population to 3–5% in the over 65 years population; whereas globally it accounts for the 3–5.5% of the total healthcare expenditure as recently estimated [2,3]. 

Recently, clay minerals have been proposed in the biomedical field in tissue engineering as enhancers of cell attachment, proliferation and differentiation [4,5], and also as antimicrobials [6]. In particular, montmorillonite (MMT, M_x_ (Al_2_-yMg_y_) Si_4_O_10_ (OH)_2_ nH_2_O) and halloysite (HNT, Al_2_O_3_ 2SiO_2_ 2H_2_O) have been recently described as biocompatible and as proliferation enhancers [7]. Both MMT and HNT are phyllosilicates, having a planar and rolled structure, respectively. Nanocomposites, based on these biomaterials, have been developed to tune cell adhesion and their biocompatibility [8,9,10,11]. The cell interaction with clays remain unclear and not fully understood, and only a few recent studies have attempted to shed light on this interaction [7,12].

However, there are many evidences in literature related to the combination of nanostructured materials with nanoscale fabrication processes, to achieve high levels of morphological control, surface and mechanical properties [13,14,15]. In light of these, layered silicates are characterized by a high aspect ratio with the ability to confer high strength to 3D structures. Moreover, unlike most inorganic fillers, layered silicates are hydrophilic, and capable to interact with the polymer matrix, by changing surface tension, conductivity, and shear viscosity. Thus, the combination of clay minerals and nanofibrous scaffolds should lead to 3D architectures which facilitate cell homing, but also enhance the cell attachment and proliferation thanks to the enhancing properties provided by biopolymers. In particular, chitosan and chondroitin sulfate are polysaccharides capable to aid cell proliferation, and moreover, the latter is also providing protection against growth factor degradation by electrostatic interaction [16]. Having previously assessed the scaffold composition in the basic biopolymers [16]; these made of pullulan, chitosan, and chondroitin sulfate; the manufacturing processing was then addressed by means of electrospinning in a one-pot process to obtain a nanofibrous scaffold which proved to enhance the mechanical properties of the scaffold.

Given the advances achieved on these materials and their manufacturing processes, the aim of this work was the design, development and characterization of electrospun 3D scaffolds, entirely based on biopolymers, loaded with MMT or HNT, as a dermal substitute for skin reparation and regeneration tested in a preclinical model, leading to tissue reparation towards a complete skin restore. 

## 2. Materials and Methods

### 2.1. Materials

The following polysaccharides were used: chitosan (CH) (β-(1-4)-linked d-glucosamine and N-acetyl-d-glucosamine) low MW 251 kDa, deacetylation degree 98%, (ChitoClear, Iceland); chondroitin sodium sulfate (CS) (β-1,4-linked d-glucuronic acid and β-1,3-linked N-acetyl galactosamine) bovine 100 EP, low MW 14 kDa, and a mixture of chondroitin A (chondroitin 4 sulfate) and chondroitin C (chondroitin 6 sulfate) (Bioiberica, Italy); pullulan (P) (based on maltotriose repeating units, linear α 1–4 and α 1–6 glucan, produced by *Aureobasidium pullulans*) low MW ~200–300 kDa (food grade, Hayashibara, Giusto Faravelli, Italy). Citric Acid (CA) (monohydrated citric acid, EP grade, Carlo Erba, I) was used as the crosslinking agent. Pharmaceutical grade clay minerals were considered: montmorillonite (MMT) (particle size: 1352 nm (±17); polydispersity index: 0.696 (±0.184)) (Veegum^®^ HS, Vanderbilt, Nashville, TN, USA) or halloysite (particle size: 563 nm (±70); polydispersity index: 0.647 (±0.077); internal diameter = 28 ± 5.1 nm and external diameter = 70 ± 8.3 nm) (Halloysite Nanotubes—HTNs) (Sigma-Aldrich, St. Louis, MO, USA).

### 2.2. Methods

#### 2.2.1. Preparation of Polymeric Blends

**Figure 1:**
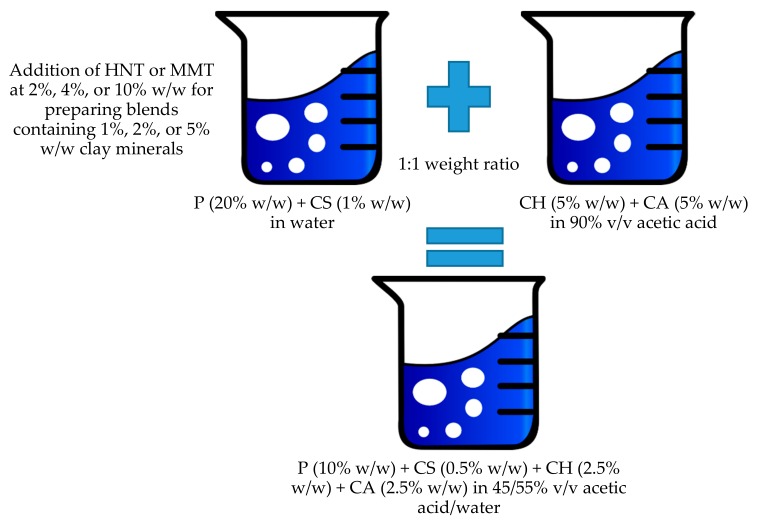
Schematic of the blend preparation.

P and CS were solubilized in water while the CH solution was prepared in 90% v/v acetic acid and CA was added. A polymeric blend was prepared by mixing P and CS with a CH solution at a 1:1 weight ratio. The preparation schematic is reported in Figure 1. 

The hybrid blends were then prepared by the addition of MMT or HNT to the polymeric blend, prepared as previously described [16]. For this purpose, clay minerals were grounded in a mortar and sieved with a 75 μm sieve. Either MMT or HNT were added to the P and CS blend at different concentrations. All the blends prepared had the same composition in polysaccharides while they were based on different concentrations of clay minerals. The composition of all the systems prepared is reported in Table 1.

#### 2.2.2. Characterization of Polymeric Blends

The surface tension of the blends was measured at T = 30 °C with a tensiometer (DY-300, Kyowa, Japan) (measurement range 0–300 mN/m) equipped with a platinum plate of 2.5 cm × 1 cm.

The electrical conductivity was determined by a conductometer (FiveGoTM-Mettler Toledo, Italy) equipped with the LE703-IP67 sensor. 

The penetrometry was measured using the Texture Analyzer TA-XT plus (ENCO, Italy), equipped with an A/TG measuring system and a 5 kg load cell. The analysis was performed employing a Perspex 20 mm cylinder probe (P/20P; Batch N° 11434). The measuring probe was lowered at a 0.50 mm/s speed up to a 3 mm penetration distance. The penetration force was recorded as a function of probe displacement.

#### 2.2.3. Preparation of Electrospun Scaffolds

Scaffolds were obtained using an electrospinning apparatus (STKIT-40, Linari Engineering, Italy), equipped with a high-voltage power supply (40 kV), a volumetric pump (Razel R99-E), a 10 mL syringe, and a conductive static collector, covered by aluminum foil. The following parameters were used: ΔV (voltage) = 22 kV, collector spinneret distance = 24 cm, polymeric solution flow = 0.4 mL/h, spinning time = 1.30 h, temperature = 30 °C, relative humidity = 30%; and needle dimensions: 0.5 × 20 mm for MMT and 0.4 × 20 mm for HNT. The obtained scaffolds were then crosslinked by heating at 150 °C for 1 h, to prevent their solubilization in aqueous media and to allow cell homing. The heating process is also reported as able to dry sterilize the products [17].

#### 2.2.4. Scaffold Characterizations

##### Chemico-Physical Characterization

Scaffold morphology was assessed by means of SEM (Tescan, Mira3XMU, CISRIC, University of Pavia) after graphite sputtering. The scaffolds were analyzed before and after the crosslinking procedure and after 6 days of hydration in distilled water. Nanofiber diameters and pores sizes were measured by image analysis software (DiameterJ plugin, Image J, NIH).

X-ray powder diffraction (XRPD) analysis was carried out using a diffractometer (X-Pert Pro model, Malven Panalytical, Italy) equipped with a solid-state detector (X-Celerator) and a spinning sample holder. The diffractogram patterns were recorded using random oriented mounts with CuKα radiation, operating at 45 kV and 40 mA, in the range 4–60° 2θ. The diffraction data were analyzed using the XPOWDER^®^ software (www.xpowder.com).

Fourier-transform infrared spectroscopy (FT-IR) spectra were recorded using spectrophotometer (JASCO 6200) with a Ge ATR. All samples were analyzed from 400 to 4000 cm^−1^ with a resolution of 0.25 cm^−1^ and the results were processed with Spectra Manager v2 software.

Thermogravimetric analysis (TGA) (TGA-50H, Shimadzu, Kyoto, Japan) was performed using a vertical oven and a precision of 0.001 mg. Approximately 40 mg of each sample were placed in aluminum pans. The experiments were performed at the 30–950 °C range and using a 10 °C/min heating rate. Additionally, differential scanning calorimetry (DSC) analyses were performed (Mettler Toledo, Columbus, OH, USA) using aluminum crucibles, a 30–400 °C temperature range, at a heating rate of 10 °C/min. All the analyses were performed in atmospheric air. 

High-resolution Transmission Electron Microscopy (TEM) was performed by means of an analytical electron microscope (AEM) (Titan G2 60–300, FEI Company, Thermo Fisher Scientific, Waltham, MA, USA) with a SUPER-X silicon-drift windowless energy dispersive X-ray spectroscopy detector. X-ray chemical element maps were also collected. The samples were directly deposited onto copper grids (300 mesh coated by formvar/carbon film, Agar Scientific, Italy).

##### Mechanical Properties

Mechanical properties of nanofibrous scaffolds were measured using a TA-XT plus Texture Analyzer (Stable Microsystems, ENCO, Italy) equipped with a 5.0 kg load cell. Before testing, nanofibrous scaffolds were cut 30 × 10 mm and the strips (thickness ranging from 150 to 200 μm, thickness gauge apparatus, Mitutoyo) were clamped between two tensile grips (A/TG probe) setting an initial distance between the grips of 10.0 mm. Mechanical properties were evaluated in the dry and hydrated state. The hydration was performed by dipping the scaffolds in water up to complete hydration (1 h). Then, the upper grip was moved forward at a constant speed of 5.0 mm/s up to break. The force at break was recorded (force at break) (TS, N/mm^2^) and the elongation (%) was calculated as follows:

E% = 100 × (L_break_ − L_0_)/L_break_ (1)

where L_break_ is the distance of the two grips at scaffold breaking and L_0_ is the initial distance of the two grips.

Moreover, Young’s Modulus (mN/cm^2^) was calculated as the slope of the initial linear portion of force vs. grip displacement [10,18,19]. 

##### Fibroblasts Biocompatibility and Adhesion

NHDFs (normal human dermal fibroblasts from juvenile foreskin, Promocell WVR, Italy) were grown with Dulbecco’s Modified Eagle Medium (Sigma, I) supplemented with 10% fetal bovine serum (FBS, Sigma, Italy) and with 200 IU/mL penicillin/0.2 mg/mL streptomycin (Sigma-Aldrich, Italy), kept at 37 °C in a 5% CO_2_ atmosphere with 95% relative humidity (RH).

Preliminarily, the cytocompatibility and proliferation in the presence of pure components were assessed. Fibroblasts were seeded with a seeding density of 25 × 10^3^ cells/well in 96-well plates. After 24 h of growth (at sub-confluence), the following samples (in growth medium, GM) were considered: CS (0.08 mg/mL), P (1.5 mg/mL), CA (0.4 mg/mL), CH (0.4 mg/mL), MMT (MMT1: 0.2 mg/mL; MMT2: 0.8 mg/mL; MMT5: 1.2 mg/mL), and HNT (HNT1: 0.2 mg/mL; HNT 2: 0.8 mg/mL; HNT5: 1.2 mg/mL). Valinomycin (Val, Fisher Scientific, Ireland) (final concentration 120 μM) was used as the cytotoxic control and GM (growth medium) as the biocompatible control.

After 24 or 72 h of contact, the MTT test was performed. Briefly, the MTT test evaluates the activity of mitochondrial dehydrogenase of vital cells that convert MTT into formazan salts. The MTT was solubilized in PBS at a concentration of 5 mg/mL per well. A total of 50 μL of the MTT solution and 100 μL of the DMEM (DMEM w/o phenol red, Sigma, Italy) were dispensed into each well and subsequently the plates were placed in an incubator at 37 °C for 3 h. The reagent was then removed from each well and the cells were washed with 150 μL of PBS to remove the samples and the un-reacted MTT solution. After PBS removal, 100 μL of DMSO were added to each well and the absorbance was detected with an ELISA plate reader (ELISA plate reader, Biorad, Italy; Epoch, Microplate Spectrophotometer, BioTek, Ireland) at a wavelength of 570 nm with a wavelength of reference of 690 nm.

Subsequently the scaffold cytocompatibility was assessed. For this purpose, scaffolds were cut to have an area of 0.36 cm^2^ to cover the bottom of a well in a 96 well-plate and fibroblasts were seeded onto each scaffold with 35 × 10^3^ cells/well and grown for 3, 6, and 10 days. An MTT assay was performed, as previously described. In addition, SEM and CLSM analysis were performed to visualize the fibroblasts adhered and proliferated to each scaffold.

Fibroblasts grown onto the scaffolds were fixed with a 3% glutaraldehyde solution for 1 h at 4 °C (glutaraldehyde 50%—Sigma Aldrich, Italy), and washed twice with PBS. As for SEM, scaffolds were dehydrated in increasing concentrations of ethanol, placed onto stub and sputtered with graphite. The images were acquired at a high voltage of 8 kV, in high vacuum, at room temperature and different magnifications (5.00 kX; 10.00 kX; 20.00 kX) (SEM: Tescan, Mira3XMU, CISRIC, University of Pavia).

As for CLSM the fixed scaffolds were stained by dipping the scaffolds in contact with 50 μL of phalloidin Atto 488 (50 μg/mL in PBS) (Sigma Aldrich, Italy) for 40 min. Cell nuclei were subsequently stained by dipping the scaffolds in 100 μL of Hoechst 33258 solution (0.5 µg/mL in PBS) (Sigma Aldrich, Italy) for 10 min. Subsequently, the samples were washed twice for 10 min with PBS and placed on microscope slide and analyzed by using a CLSM (Leica TCS SP2, Leica Microsystems, Italy) using λex = 346 nm and λem = 460 nm for Hoechst 33342 (Sigma, Italy) and λex = 501 nm and λem = 523 nm for phalloidin Atto 488 (Sigma, Italy). 

##### Cytocompatibility of Macrophages and Pro-Inflammatory Immune Response

Human monocytic cell line THP-1 (American Type Culture Collection, Manassas, VA, USA) was cultured in RPMI-1640 medium (Gibco, Thermo Fisher, Ireland) supplemented with 10% fetal bovine serum (FBS, Sigma, Ireland), and with 200 IU/mL penicillin/0.2 mg/mL streptomycin kept at 37 °C in a 5% CO_2_ atmosphere with 95% relative humidity (RH). A total of 1 × 10^5^ cells/mL were treated with 100 nM phorbol-12-myristate-13-acetate (PMA, Sigma Aldrich, Germany) for 48 h. After 48 h the cells were differentiated into macrophages and let to rest for 24 h before being treated. 

Preliminarily, the cytocompatibility of the pure components was assessed considering sample concentrations as previously described in Section “Fibroblasts Biocompatibility and Adhesion” paragraph. Valinomycin (Val, Fisher Scientific, Ireland) or cysplatinum (Cys, Sigma Aldrich, Ireland) (final concentration 120 μM) was used as the cytotoxic control and GM (growth medium) as the biocompatible control.

THP-1 was differentiated using PMA and 20 × 10^3^ cells were seeded in each well of the 96-well plates. After 24 h rest, the components were added to each well and the biocompatibility was assessed after 24 or 72 h of contact time, using MTT test, as previously described.

Subsequently, the scaffold cytocompatibility was assessed. Scaffolds were cut to have an area of 7.65 cm^2^ (diameters of 1.5 cm) and placed on a cell crown (Sigma, Italy). THP-1 cells were differentiated by seeding 200 × 10^3^ cells on the bottom of each well of the 24-well plates. After 24 h rest, the scaffold placed on the cell crown was inserted in the well. The cytocompatibility was assessed using an MTT assay (Sigma Aldrich, Ireland) after 24 or 72 h of contact time, as previously described. 

TNF-α, pro-inflammatory cytokine, was assayed to evaluate the pro-inflammatory immune response using the commercially available ELISA kit (BioLegend, Medical Supply Co. Ltd., Ireland). Supernatants were collected from the cultures at 24 or 72 h after the treatment with the components or the scaffolds. 

The cytokine secretion by macrophages was assayed at 450 nm with 570 nm as the reference wavelength (Epoch microplate reader, Biotek, Mason Technologies, Ireland). The method was linear in the concentration range from 7.8 to 500 pg/mL with the R^2^ always higher than 0.995. Lipopolysaccharide (LPS, 100 ng/mL for 24 h) was used as the positive control.

#### 2.2.5. Statistical Analysis

Statistical differences were evaluated using a non-parametric test: the Mann–Whitney (Wilcoxon) W test, (Statgraphics Centurion XV, Statistical Graphics Corporation, MD, USA). Differences were considered significant at *p* < 0.05.

## 3. Results and Discussion

### 3.1. Polymeric Blend Characterization

The characterization of the polymers in terms of conductivity (µS/cm), surface tension (N/m), and consistency (mN × mm) of all the polymeric blends is reported in Table 2. 

The increase in clay mineral concentration caused an increase in conductivity, surface tension, and consistency. In particular, MMT at concentrations higher than 1% increased conductivity, surface tension, and consistency to values greater than those of the blank (blend without MMT). This behavior was less evident when HNT was blended with the polymeric mixture, probably due to the different particle sizes of the two clays. It is conceivable that the addition of HNT or MMT to the polymer blend caused a partial immobilization of the polymer chains due to charge–charge or hydrophobic interaction. In case of a lower amount of clay minerals added (1%), this determined a decrease in consistency and conductivity, while higher amounts (2% or 5%) had the opposite behavior probably due to an excess of charges from MMT or HNT not counterbalanced from the polymers in the solution [14].

### 3.2. Scaffold Characterizations

#### 3.2.1. Chemico-Physical Characterization

**Figure 2:**
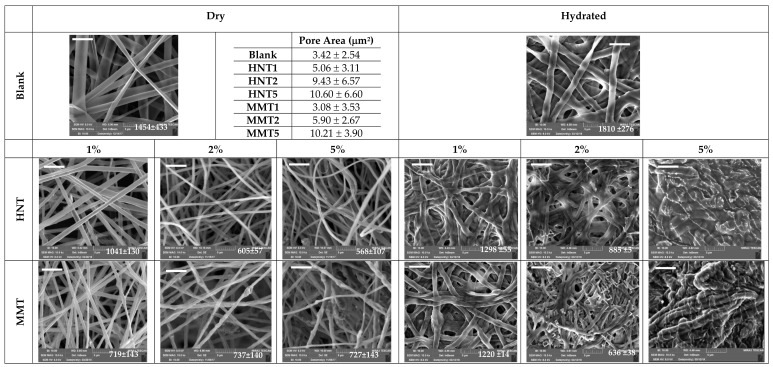
SEM images of the scaffolds: blank (scaffold without clay) and montmorillonite (MMT) or halloysite (HNT) scaffolds containing 1%, 2%, or 5% of the clay mineral in the dry and hydrated state (the bar in each image is 5 μm). In the inset the fiber diameters (nm) are reported (mean values ± SD; n = 100).

Advanced images such as SEM microphotographs of MMT- or HNT-based scaffolds and a blank scaffold in the dry or hydrated state are presented in Figure 2. The pore size evaluated for each scaffold is reported in an inset. Furthermore, in each image, the fiber diameter is reported.

In the dry state, the blank scaffold was characterized by fibers with a smooth surface, and fiber diameters with a coarse distribution around 1500 nm. HNT scaffolds were therefore characterized by the nanofibers’ regular structure and smooth surface, where the addition of the clay minerals in the scaffolds provided a significant decrease in the fiber dimensions. The scaffolds containing 2% and 5% HNT had halved the diameter size compared to the blank scaffolds. Moreover, HNT determined a much more regular structure compared to the blank scaffolds. This was probably due to the unique structure of HNT, which are nanotubes with a high aspect ratio of 10 [9,20], thus capable of aligning along the fiber length and providing increasing surface tension. This allowed to obtain a more regular polymer solution jet during the electrospinning process. MMT scaffolds were characterized by nanofiber portions with a regular, smooth surface spaced out in a broaden interwoven, resembling knots, and with a wider structure organisation. These conceivably could be related to montmorillonite [9,21]. The increase of MMT concentration, especially in the 5% MMT scaffold, caused an increase in the surface roughness of the fibers, while the fiber diameters were significantly lower than those of the blank scaffold, although this was not influenced from the clay concentration. It is reported that clay minerals could act as a compatibilizer and this could positively affect the electrospinning of a polymer blend, containing positively and negatively charged polymers, as chitosan and chondroitin sulfate. Therefore, MMT or HNT could conceivably reduce the interfacial tension in the polymer blend, thus facilitating electrospinning, to obtain finer and more homogeneous nanofibers with respect to the blank [22]. Moreover, it is reported that the conductivity of the solution, influenced by the clay content, could increase the charge on the surface of the droplet to form a Taylor cone, and consequently could cause the decrease in the fiber diameter [23].

The presence of HNT or MMT in the scaffolds increased the systems porosity, and although there were not significant differences, the increase of clay mineral concentration increased the pore dimensions: this seems inversely related to the decrease of fiber dimensions. Porosity and fiber dimensions seem to have a crucial role for facilitating cell adhesion in the scaffold: The porosity could convert the scaffold from a surface to a fiber network, which could act as a sieve to the home cells.

The hydration significantly increased the fiber dimension, however no solubilization of the scaffold occurred thanks to the cross-linking by heating: The structural analysis (FTIR and SAXS) and the water holding capacity suggested that no new chemical bond was formed upon heating treatment while a polymer chain felting occurred when water was released due to thermal treatment, resulting in local physical multi-entanglement between the fibers, which could not be released by simple hydration. When HNT or MMT were at lower concentrations, up to 2%, the hydration did not alter the fibrous structure of the scaffolds, while when HNT or MMT were at a 5% concentration, the fibers were fused although the morphology was preserved. The higher content of hydrophilic clay minerals could weaken the overall scaffold structure, since the polymer chains in the matrix loosened their tightness causing a higher fiber swelling. 

**Figure 3:**
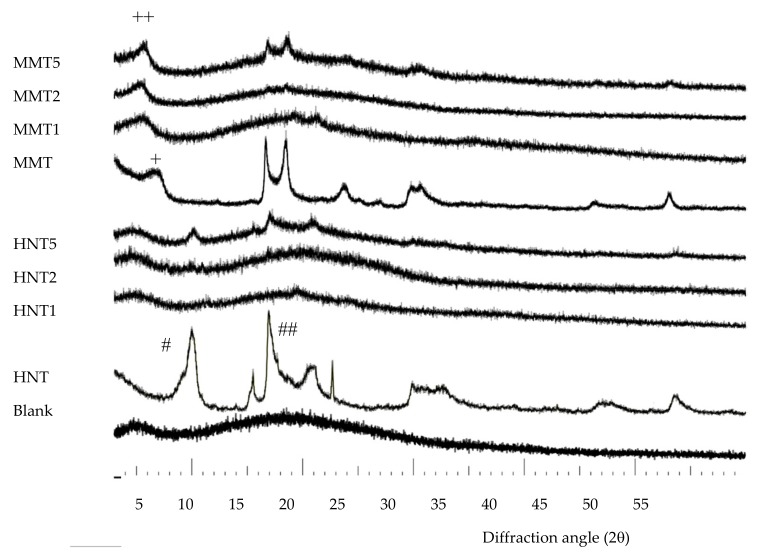
Comparison of XRPD patterns of all the scaffolds developed against pristine HNT and MMT.

XRPD of all the scaffolds developed is presented as comparison to the pristine HNT and MMT, in Figure 3. A blank scaffold was characterized by an amorphous pattern and no crystalline or paracrystalline behavior could be detected. Pristine HNT was characterized by a peak at 12.25° 2θ corresponding to 7.24 Å, a typical height of the dehydrated HNT interlaminar spaces (Figure 3, peak labelled as #). The peaks at 20.14° 2θ and at 25.03° 2θ confirmed the HNT tubular structure and its phyllosilicate nature (Figure 3, peak labelled as ##) [24]. HNT-loaded scaffolds were characterized by patterns more similar to that of the blank scaffold rather than those of pristine HNT: In these patterns, only the peaks attributable to the phyllosilicate nature of HNT (peaks at 25.03° 2θ) were present and there was a signal increase directly related to HNT concentration. Since the diffraction angle remained constant in all the patterns and it was the same as in the pristine HNT, it could be argued that no enlargement of the interlaminar space of the rolled structure occurred.

The pattern of pristine MMT was characterized by a peak at 7° 2θ due to the distance of the d001 basal reflection, corresponding to 12.2 Å, characteristic of predominantly Na+ smectites (Figure 3 peak labelled as +).

In the MMT scaffolds, the d001 basal reflection was shifted to approximately 6° 2θ. This corresponded to a distance of 14.0 Å, suggesting that there was an enlargement of the interlayer space (Figure 3, peak labelled as ++). This was associated with the intercalation of the biopolymer into MMT layers, probably as monolayer between the silicate layers [10,25]. 

**Figure 4:**
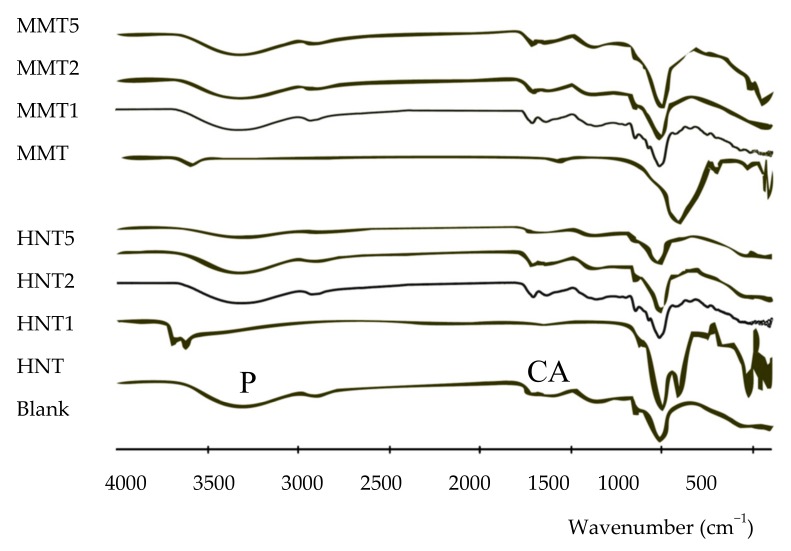
Comparison of the FTIR spectra of all the scaffolds developed against to the pristine HNT and MMT. In the blank spectrum the signals related to P (pullulan) and CA (citric acid) are marked.

FTIR spectra of all the scaffolds developed is presented as comparison to the pristine HNT and MMT in Figure 4. In the spectrum of the blank scaffold, the signals related to pullulan (P) and citric acid (CA) are marked. These characteristic signals were present also in all the scaffolds containing either MMT or HNT.

HNT spectrum was characterized by two signals at 3696 cm^−1^ and 3622 cm^−1^, due to OH inner and outer stretching, respectively, while the MMT spectrum was characterized by a signal at 3624 cm^−1^ caused by the Al–OH stretching. The characteristic peaks of both the clay minerals were hidden by a broad band due to a typical polysaccharide signal (hydrogen bonds of –OH and –NH_2_ groups). (pullulan: 3331 cm^−1^ and chitosan: 3355 cm^−1^). Moreover, the vibrational band of NH_3_^+^ groups of chitosan could be identified at 1550 cm^−1^, as a shoulder [26,27]. 

**Figure 5:**
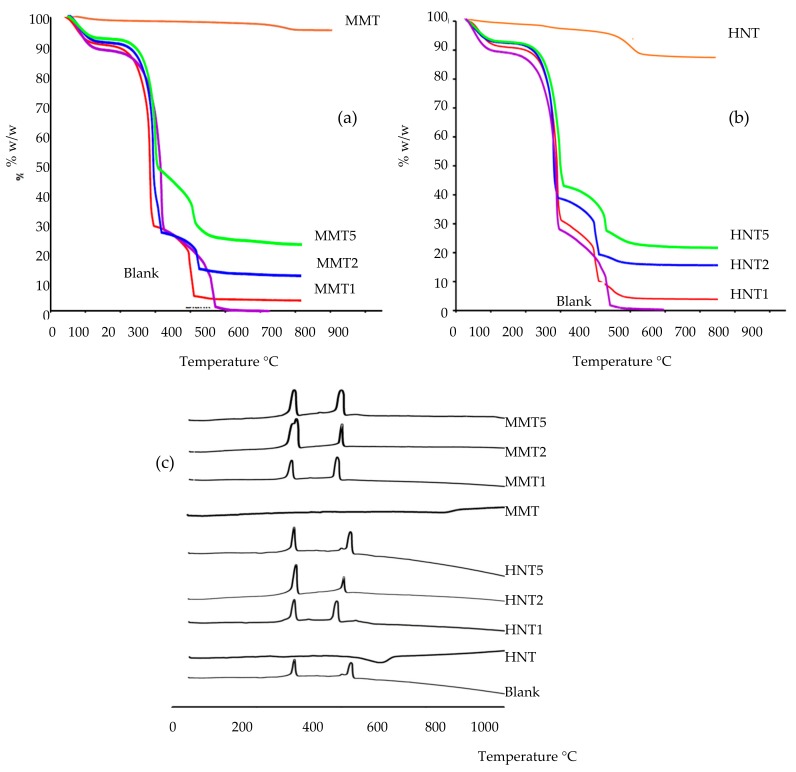
Comparison of TGA (**a**,**b**) and DSC (**c**) profiles of all the scaffolds against pristine HNT and MMT.

TGA (a,b) and DSC (c) profiles of all the scaffolds, compared to pristine HNT and MMT is reported in Figure 5. Thermal analysis was performed to characterize the role of the clay minerals in the scaffold structure. TGA and DCS profiles suggested than both HNT and MMT had high thermal stability. 

TGA analysis suggested that all the scaffolds, independently of the clay mineral loaded and its concentration, were subjected to a slight weight loss corresponding to the evaporation of hydration water. This accounted for about 7% of the scaffolds weight (30–101 °C) (Figure 5a). DSC analysis (Figure 5b,c) showed a slight endothermic event between 30 and 110 °C, confirming the TGA results. 

Additionally, characterization showed that all the scaffolds reported a more prominent weight loss (onset: about 230 °C; offset: about 400 °C) with greater mass loss to reach 26%, 12%, and 7% of residual weight for the MMT 5, MMT2, and MMT1, respectively. These coincided with two endothermic events in the DSC thermograms and these could be conceivably caused by the decomposition [28,29]. 

The clay minerals loaded into the scaffolds, independently from the types and concentrations used, maintained their thermal stability and were able to slightly stabilize the scaffolds towards thermal degradation, increasing the onset temperatures of each thermal event; this is particularly evident in the TGA profiles (Figure 5a,b).

The residual mass was related to the clay mineral concentration in each scaffold: HNT: 5.56% for 1% loading; 11.40% for 2%, and 21.02% for 5%; MMT: 5.46% for 1% loading; 11.02% for 2%, and 22.36% for 5%. 

**Figure 6:**
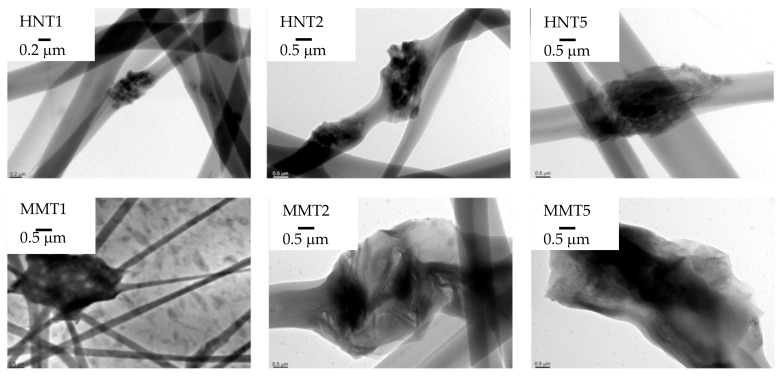
HRTEM microphotographs of the broadened parts of the fibers.

**Figure 7:**
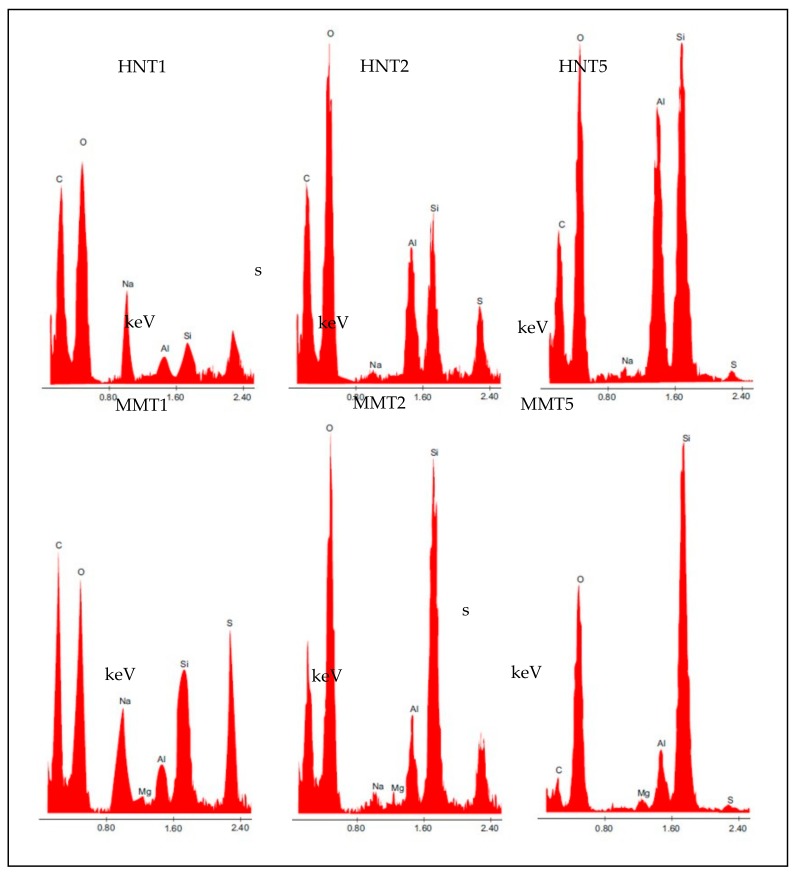
EDX spectra of the broadened parts of the fibers.

HRTEM microphotographs of the broadened parts of the fibers is presented in Figure 6, and Figure 7 reports their EDX spectra.

The HRTEM and EDX analysis evidenced that the broadened parts were based on clay mineral particles: The tubular structure of HNT and laminar one of MMT could be identified. Moreover, the elemental analysis showed the presence of Al and Si typical in the case of HNT and the presence of Al, Si, and Mg in the case of MMT, but also of S and C, to indicate that the inorganic material was embedded into the organic component. 

#### 3.2.2. Mechanical Properties

**Figure 8:**
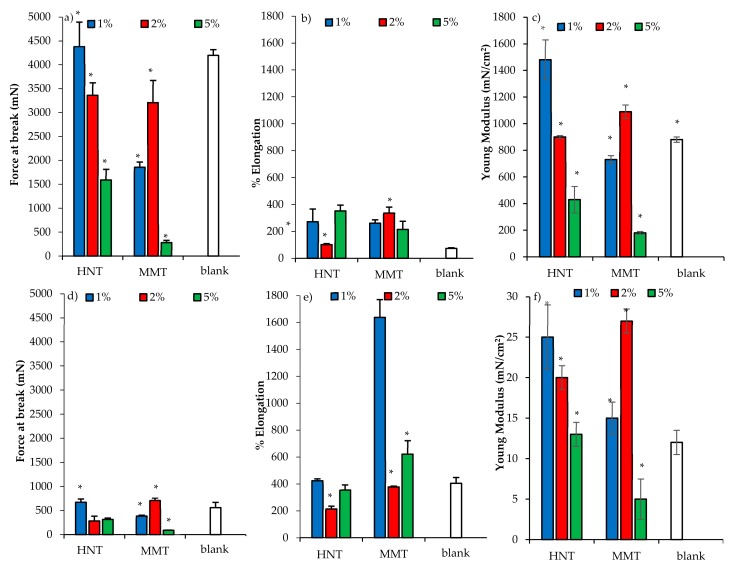
Mechanical properties (force at break mN, **a**–**c**; elongation %, **b**–**e**; Young’s Modulus mN/cm^2^, **c**–**f**) for dry (**a**–**c**) and wet (**d**–**f**) scaffolds loaded with HNT or MMT at different concentrations (mean values ± SD; n = 3). Statistics: * = Mann–Whitney (Wilcoxon) W test *p* < 0.05.

The mechanical properties (force at break mN, a,b; elongation %, c,d; Young’s Modulus mN cm^2^, e,f) of scaffolds loaded with HNT or MMT, in dry (a,c,e) or wet (b,d,f) conditions are presented in Figure 8. 

In the dry state, the increase of HNT caused a decrease of force at break (Figure 8a) and of system elasticity (Figure 8c), while MMT reinforced the scaffold structure increasing the resistance to break (Figure 8a) and the system elasticity (Figure 8c) up to the 2% concentration; a further increase weakened the scaffold. The scaffolds were characterized by moderate deformability higher than that of the blank scaffolds (Figure 8b). The hydration caused a remarkable decrease in resistance to break, an increase of deformability, and a loss of elasticity (Figure 8d–f). Clay minerals seem to reinforce the scaffold structure; however, if their concentration exceeded a certain threshold, the presence of particles embedded into the polymeric matrix could disrupt the polymer chain entanglements, weakening the scaffolds.

#### 3.2.3. Fibroblasts Biocompatibility and Adhesion

**Figure 9:**
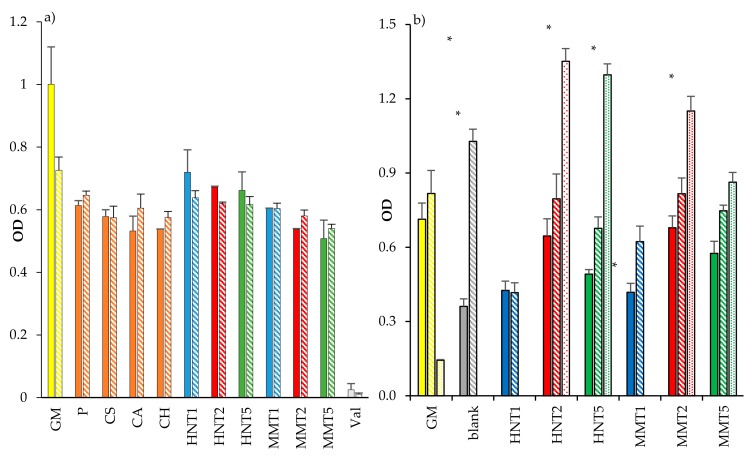
Cytocompatibility (OD, optical density) towards fibroblasts of (**a**) the scaffold components (GM: growth medium; P: pullulan; CS: chondroitin sulfate; CA: citric acid; CH: chitosan; Val: valinomycin) after 3 days (plain color) and 6 days (oblique line) of growth, and (**b**) of the scaffolds loaded with HNT or MMT after 3 days (plain colors), 6 days (oblique lines), and 10 days (spotted) of growth (**b**) (mean values ± SD; n = 8). Statistics: * = Mann–Whitney (Wilcoxon) W test *p* < 0.05.

Assessment for the cytocompatibility (OD, optical density) towards fibroblasts of (a) the scaffold components after 3 and 6 days of growth, and (b) the scaffolds after 3, 6, and 10 days of growth is summarized and presented in Figure 9. All the scaffold components (solubilized or dispersed in growth medium, GM) were characterized by similar biocompatibility considering 3 or 6 days of interaction, attachment and exposure with the fibroblasts (Figure 9a). Both HNT and MMT showed good biocompatibility. Of note, the fibroblast cytocompatibility decreased with their concentrations: in these conditions, clay minerals as powders (not soluble in GM) could negatively influence cell viability due to their sedimentation. 

Fibroblast cells showed to grow onto the scaffolds, and these were compared to those of the control (GM, cell growth in standard conditions) (Figure 9b). After 3 days of cell growth onto the scaffolds loaded with either 2% HNT or 2% MMT, these were able to proliferate similarly to that seen when in simple substrate exposed to growth medium (as reference control). Conversely, all the other compositions caused a significant decrease in cell viability, as shown in Figure 9a,b. After 6 days, the blank scaffold and the scaffolds loaded with MMT (at all the concentrations) and HNT (at 2% and 5%) enhanced cell proliferation similarly to control samples, and only the scaffolds loaded with HNT at 1% was not able to show any proliferation as the control. After 10 days, the GM was unable to progress into further cell growth due to the extensive cytotoxicity issues. Nonetheless, the scaffolds loaded with both the clay minerals at 2% and 5% showed increased cell proliferation, and HNT and MMT at 2% showed the best proliferative responses. A possible explanation could be due to the specific properties of halloysite and montmorillonite which showed the enhancing fibroblast proliferation [9,10].

**Figure 10:**
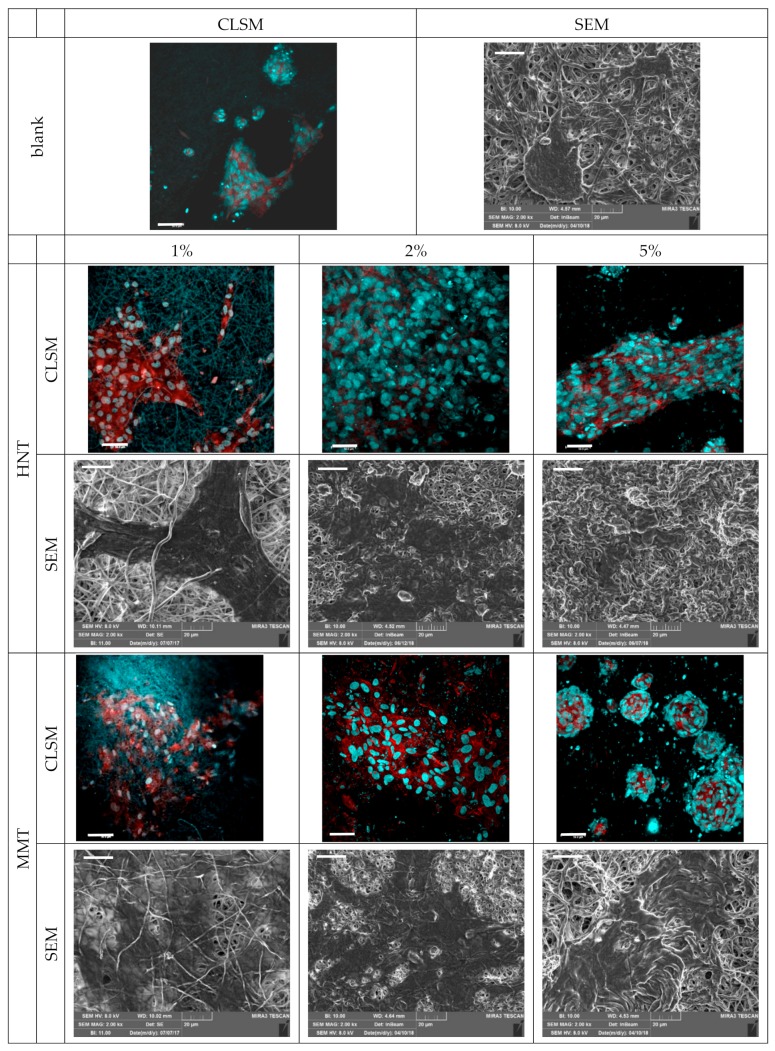
CLSM (scale bar: 50 μm) and SEM (scale bar: 20 μm) representative images of fibroblasts grown for 6 days onto the scaffolds loaded with HNT or MMT at 1%, 2%, and 5% (CLSM: in blue: nuclei; in red: cytoskeleton). Cell proliferation pattern showed by the nuclear staining increased frequencies in the HNT and MMT.

These results are in agreement with the analysis carried out on the observed CLSM and the SEM images (Figure 10). In particular, from the SEM and CLSM analysis it could be suggested that the loading of clay minerals in the scaffolds allowed homogeneous fibroblast attachment, spreading and growth all over the scaffolds. Interestingly, only the scaffold containing MMT at 5% caused cell growth in clusters and this could be associated with the irregular surface and morphology of the scaffold fibers, which prevented cell attachment and surface adhesion. However, the scaffolds loaded with HNT or MMT at 2% allowed the fibroblasts to maintain their fusiform structure and aligned and elongated the cytoskeleton filaments, enhancing cell confluency.

In a previous work [16], a blank scaffold was characterized for surface zeta potential by means of the measurements of a streaming current and streaming potential. Such a scaffold possessed 2.9 isoelectric point with a zeta potential plateau above pH 5, at about −13.8 mV, and this was related to the strong interaction between CS and the amino groups of CH. Moreover, the structural features at the mesoscale were characterized by means of SAXS analysis [16]. These evidenced that nanofibers in the scaffold were characterized by tubular structures and that the hydration caused polymer chains protruding and stretching out from the fibers surface. The scaffold swelling was due to the dilatation of the scaffold mesh rather than single fiber swelling [16]. In addition, the blank scaffold possessed a certain degree of antibacterial activity against *Staphylococcus aureus*, and this was attributable to chitosan that retained its antimicrobial properties although entangled in the scaffold structure. Furthermore, the blank scaffold demonstrated to be resorbed in vivo in a preclinical (burn excisional murine) model after the lesion healing [16]: the evaluation of the degradation pathway evidenced that lysozyme, continuously secreted by white cells (macrophages and neutrophils) during the inflammatory phase of wound healing, played a crucial role in scaffold degradation [30]. 

#### 3.2.4. Cytocompatibility of Macrophages and Pro-Inflammatory Immune Response

**Figure 11:**
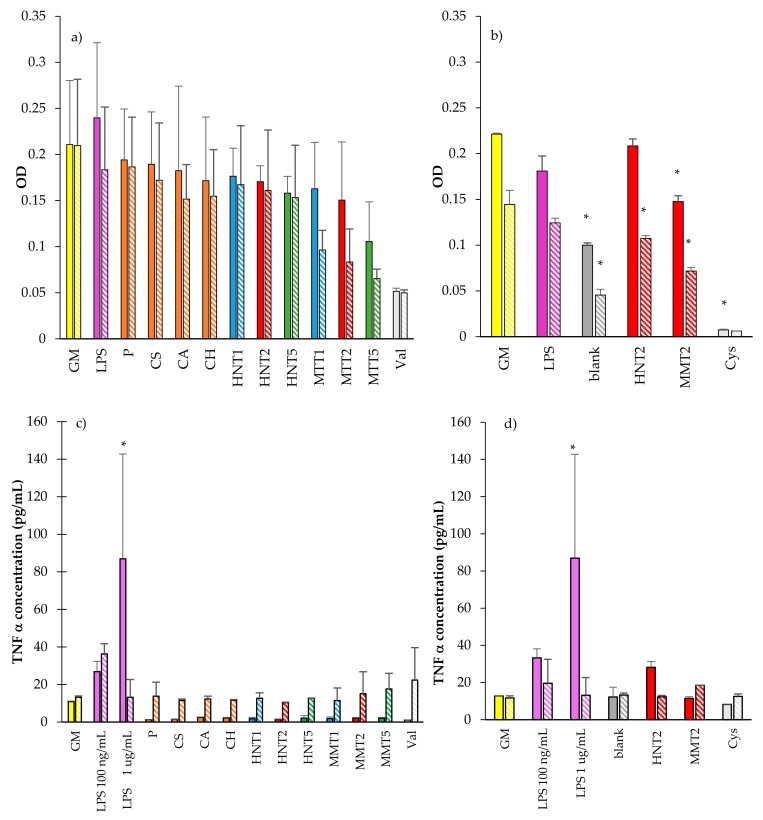
Cytocompatibility of THP-1 macrophages of the scaffold components (**a**), and the scaffolds after 24 (plain color) and 72 h (oblique lines) of exposure and contact (**b**). TNFα cytokine expression and concentrations (pg/mL) for THP-1 cells exposed to components (**c**); and (**d**) scaffolds (mean values ± SD; n = 8) (GM: growth medium; LPS: lipopolysaccharide; P = pullulan; CS: chondroitin sulfate; CA: citric acid; CH: chitosan; HNT1: 1% w/w halloysite; HNT2: 2% w/w halloysite; HNT5: 5% w/w halloysite; MMT1: 1% w/w montmorillonite; MMT2: 2% w/w montmorillonite; MMT: 5% w/w montmorillonite; blank: unloaded scaffold; Val: valinomycin; Cys: cysplatinum) Statistics: * = Mann–Whitney (Wilcoxon) W test *p* < 0.05.

Cytocompatibility was carried out by means of MTT assessment, recorded as intensity profile, analysed and presented on the macrophages in response to the scaffold components (a), the exposure and interaction with scaffolds after 24 and 72 h time points (b), as shown in Figure 11a,b. Moreover, TNFα concentrations (pg/mL), secreted by the macrophages after the contact with the components or the scaffolds are reported, in parallel to the MTT intensity readouts, in response to the components (Figure 11c); and the scaffolds (Figure 11d). From the collective results, it emerges that for all the cell substrates exposed to the components of the scaffold prepared in solution and for the LPS, at higher concentration (used as proinflammatory control) presented a full biocompatibility range when considering the two time points 24 or 72 h of contact with the macrophages (Figure 11a). Both HNT and MMT showed comparable biocompatibility although the macrophages’ cytocompatibility decreased with MMT concentrations probably due to their different degradation profile up to 72 h. 

The scaffold cytocompatibility towards fibroblasts and their proinflammatory response were evaluated only in the case of 2% clay mineral loading. In fact, the results obtained from mechanical properties and cell adhesion and proliferation capacity suggest that 2% clay mineral conferred to the scaffolds suitable stiffness/elasticity combined with the capability to support cell homing. After 24 and 72 h of growth, the scaffolds loaded with either HNT or MMT at 2% allowed macrophage viability similarly to that of their negative controls (GM, cell growth in standard conditions) (close to 75% of viability) (Figure 11b). However, the blank scaffold showed 50% cell viability with respect to the GM. The increase in the exposure time at 72 h also shows a decreased viability of the macrophages, when compared to their negative controls (GM). 

Interestingly when looking at cytokine secretion, TNFα was secreted in significantly lower amounts from the cells in exposed and in contact with scaffold components for 24 h, while after 72 h of contact, the TNFα secretions were similar to the negative control, GM. The LPS positive controls provided the evidence that the cells were responsive to stimuli (proinflammatory agent) (Figure 11c). Considering 24 h exposure, the scaffolds caused a TNFα secretion similar to that of the GM. The scaffold loaded with HNT induced the TNFα secretion similar to those obtained when LPS was at a lower concentration but significantly lower than those observed for LPS at a higher concentration (Figure 11d). After 72 h of exposure time, all the scaffolds were assessed in their TNFα secretions that were not significantly different to the GM. This could be possibly also linked to the decrease in macrophage viability after 72 h in standard growth conditions. These results showed that HNT and MMT scaffolds did not show any significant proinflammatory activity compared to controls.

## 4. Conclusions

Halloysite or montmorillonite were loaded in an electrospun polysaccharidic scaffold in a one-pot process. Halloysite scaffolds were characterized by a fiber regular structure and smooth surface, and did not show any structural alterations when embedded in the polymeric matrix, probably due to the nanotubular structure of this clay mineral. MMT scaffolds were characterized by nanofiber portions with a regular, smooth surface, spaced out in broadened parts as knots with a scattered structure, possibly due to its structure. Moreover, MMT inclusion in the polymeric matrix of the scaffold caused interlayer space enlargement, causing the biopolymer intercalation into the MMT galleries, resulting in a deep interaction between the scaffold matrix and clay mineral. HNT or MMT (2% concentration) in the scaffolds were able to sustain homogeneous fibroblast spreading all over the scaffolds and their growth up to confluency, maintaining a cell fusiform structure and aligned and elongated cytoskeleton filaments. HNT and MMT (2% concentration) scaffolds. Due to their capability to support and enhance fibroblasts proliferation with negligible proinflammatory activity, these scaffolds are promising for applications in wound healing: Their capability to enable cell attachmnent and adhesion is probably due to their morphological 3D structure-assisted cell homing, and this could facilitate wound healing in vivo.

## 5. Patents

Sandri: G.: Bonferoni, M.C.; Rossi, S.; Ferrari, F. Electrospun nanofibers and membranes, PCT/IT2017/000160, 2017.

## Figures and Tables

**Table 1:** Composition (% w/w) of the polymeric blends.

% w/w	MMT	HNT	P	CH	CA	CS	H_2_O/CH_3_COOH
Blank	-	-	10	2.5	2.5	0.5	55/45
MMT1	1	-					
MMT2	2	-					
MMT5	5	-					
HNT1	-	1					
HNT2	-	2					
HNT5	-	5					

**Table 2:** Conductivity, surface tension, and consistency of the polymer blends (blank) and polymer blends containing MMT or HNT at 1%, 2%, or 5% w/w (mean values ± SD; n = 3).

Sample	Conductivity (µS/cm)	Surface Tension (N/m)	Consistency (mN × mm)
Blank	1363 ± 11	36.6 ± 0.2	188 ± 2
HNT1s	1271 ± 3	37.7 ± 0.2	155 ± 3
HNT2s	1303 ± 4	38.1 ± 0.1	175 ± 2
HNT5s	1352 ± 9	38.6 ± 0.2	203 ± 5
MMT1s	1255 ± 23	38.7 ± 0.4	171 ± 2
MMT2s	1527 ± 17	40.8 ± 0.2	308 ± 8
MMT5s	1663 ± 9	41.3 ± 0.1	338 ± 4
